# Development of a Novel, Highly Sensitive System for Evaluating Ebola Virus Particle Formation

**DOI:** 10.3390/v17071016

**Published:** 2025-07-19

**Authors:** Wakako Furuyama, Miako Sakaguchi, Hanako Ariyoshi, Asuka Nanbo

**Affiliations:** 1 National Research Center for the Control and Prevention of Infectious Diseases, Nagasaki University, Nagasaki 852-8523, Japan; wfuruyama@nagasaki-u.ac.jp (W.F.); bb30222001@ms.nagasaki-u.ac.jp (H.A.); 2 Central Laboratory, Institute of Tropical Medicine (NEKKEN), Nagasaki University, Nagasaki 852-8523, Japan; miako@nagasaki-u.ac.jp; 3 Department of Emerging Infectious Diseases, Institute of Tropical Medicine (NEKKEN), Nagasaki University, Nagasaki 852-8523, Japan

**Keywords:** Ebola virus, VP40, HiBiT system, viral particle formation, antiviral screening

## Abstract

Ebola virus (EBOV) causes severe hemorrhagic fevers in humans, and effective countermeasures remain limited. The EBOV-encoded major matrix protein VP40 is essential for viral assembly, budding, and particle release, making it a promising target for antiviral drug development. However, no approved drugs currently target the viral particle formation process. In this study, we established a simple and highly sensitive screening system to evaluate VP40-mediated virus-like particle (VLP) formation under biosafety level −2 conditions. The system uses the HiBiT luminescence-based reporter fused to VP40, allowing for the detection of VP40 release. Our results demonstrate that the HiBiT sequence fused at the N-terminus [HiBiT-VP40 (N)] retains VP40′s ability to form VLPs, supporting its use as a functional reporter. Furthermore, we validated the system by assessing the role of Rab11-dependent trafficking in VP40-mediated budding and by evaluating the effect of nocodazole, a microtubule depolymerizer, on VLP release. This novel screening system provides a convenient and reliable platform for screening potential inhibitors targeting the late stages of EBOV infection, including viral particle formation and release. Additionally, its potential adaptability to other filoviruses suggests wide applicability in the discovery and development of additional novel therapeutic agents.

## 1. Introduction

Ebola virus (EBOV), a member of the family *Filoviridae*, causes Ebola virus disease (EVD) in humans, with a fatality rate of up to 90% [1]. The 2013–2016 EBOV epidemic in West Africa was the largest recorded outbreak, resulting in over 28,000 cases and 10,000 deaths [1,2]. The second-largest outbreak occurred in the Democratic Republic of the Congo between 2018 and 2020, with more than 3000 cases and 2000 fatalities [1]. These epidemics have accelerated efforts to develop antiviral strategies and vaccines [3,4]. However, they also highlight the limited efficacy of existing treatments and urgent need for more effective countermeasures.

EBOV has a negative-sense RNA genome encoding at least seven distinct proteins: nucleoprotein (NP), polymerase cofactor (VP35), matrix protein (VP40), glycoprotein (GP), transcription activator (VP30), minor matrix protein (VP24), and RNA-dependent RNA polymerase (L) [5]. The viral single transmembrane GP is responsible for both receptor binding and membrane fusion during entry [6], making it a key target for therapeutic intervention. Consequently, numerous countermeasures, including neutralizing antibodies targeting the GP, have been developed [7]. Another therapeutic strategy involves nucleotide analogs that inhibit RNA-dependent RNA polymerase [6]. Notably, clinical trials conducted during the EVD epidemic in the Democratic Republic of the Congo led to the approval of two neutralizing antibody-based therapies for EBOV treatment [8]. To identify additional drug candidates, various screening methods have been developed, including replication-incompetent pseudotype viruses and minigenome systems [9,10,11,12,13].

The major matrix protein VP40, the most abundant viral protein, is located beneath the viral envelope and plays a critical role in maintaining virion structural integrity and maturation [14,15,16,17,18,19]. VP40 forms a dimer, which further assembles into a flexible filamentous matrix structure [20]. Throughout the EBOV lifecycle, VP40 serves multiple functions, including virion formation, regulation of viral transcription, and the assembly and budding of mature virions. Thus, VP40 is considered a promising therapeutic target for inhibiting viral assembly and budding. However, no approved therapeutics currently target its functions.

Research involving infectious EBOV requires handling in maximum-containment laboratories under biosafety level (BSL)-4 conditions, posing considerable challenges for anti-infective drug discovery. To circumvent these challenges, several studies have identified potential drug candidates targeting EBOV VP40-mediated viral particle formation through virtual screening of large chemical databases [21,22,23]. These computational screening approaches, which rely on molecular and structural analyses, primarily focus on the specific functions of VP40, such as dimerization or viral RNA transcription. In addition to in silico approaches, a luciferase-based reporter assay was previously developed to measure VP40-mediated virus-like particle (VLP) budding by co-expressing luciferase that becomes passively incorporated into VLPs [24,25]. However, these assays may not precisely reflect VP40-driven budding activity. Moreover, cell-based assays for evaluating VP40-mediated viral particle assembly and formation by directly fusing reporter proteins with VP40 have been reported [26,27]. However, fusion of these large proteins may interfere with the dimerization of VP40. Therefore, to facilitate the development of therapeutics targeting all aspects of VP40 function, the establishment of highly sensitive and quantitative cell-based screening methods that directly evaluate the efficiency of formation of Ebola virus particle is crucial.

To address these challenges, we developed a novel EBOV VP40 detection system using the HiBiT assay under BSL-2 conditions. This system provides a practical platform for identifying antivirals that target viral assembly, budding, and release.

## 2. Materials and Methods

### 2.1. Cells

Human embryonic kidney HEK293 cells (American Type Culture Collection, Manassas, VA, USA) were grown in Dulbecco’s modified Eagle’s medium (DMEM; Wako Pure Chemical, Osaka, Japan) containing 10% fetal bovine serum (Sigma-Aldrich, St. Louis, MO, USA), 100 U/mL penicillin, and 100 μg/mL streptomycin (Wako Pure Chemical). Expi293 F cells (kindly gifted by Dr. Kentaro Yoshii, Nagasaki University, Nagasaki, Japan) were grown in Expi293 expression medium (Thermo Fisher Scientific, Waltham, MA, USA) and maintained at 37 °C in 8% CO_2_.

### 2.2. Plasmids and siRNA

HiBiT is an 11-amino-acid peptide tag that forms a functional NanoLuc complex upon interaction with its complementary fragment, LgBiT, in the presence of a substrate [28]. The HiBiT tag sequence (VSGWRLFKKIS) was fused to the EBOV VP40 gene at either the N-terminus [HiBiT-VP40 (N)] or C-terminus [HiBiT-VP40 (C)] using an In-Fusion Cloning kit (TaKaRa Bio, Kusatsu, Japan). HiBiT-VP40 L117R was generated by site-directed mutagenesis using KOD One^®^ PCR Master Mix (TOYOBO, Osaka, Japan). Each construct was inserted into an expression plasmid (pCAGGS). Detailed cloning strategies are available upon request. The pEGFP-C3 plasmids encoding enhanced GFP-fused wild-type Rab11 (GFP-wtRab11) and the dominant-negative form of Rab11 (GFP-dnRab11) were provided by Dr. Angela Wandinger-Ness (University of New Mexico, Albuquerque, NM, USA) [29]. For small interfering RNA (siRNA) treatment, target sequences corresponding to human *Rab11a* and *Rab11b* were selected and synthesized (Thermo Fisher Scientific, Waltham, MA, USA) [30].

### 2.3. Western Blot Analysis

HEK293 cells were transfected with expression plasmids for wtVP40, HiBiT-VP40 (N), HiBiT-VP40 (C), or HiBiT-VP40 L117R using TransIT-X2 (Mirus Bio, Madison, WI, USA). At 48 h post-transfection (h.p.t.), the culture supernatants were harvested and centrifuged at 440× *g* for 5 min at 4 °C to remove cell debris. The supernatants and whole-cell lysates from each sample were incubated in Laemmli sample buffer for 5 min at 95 °C, followed by Western blotting. Proteins were detected using mouse monoclonal antibodies against EBOV VP40 (cl. 3G5; IBT Bioservices, Rockville, MD, USA; 1:1000 dilution), GP (cl. ZGP12/1.1; kindly provided by Dr. Ayato Takada, Hokkaido University, 1 μg/mL), NP (cl. 7–71.5, kindly provided by Dr. Yoshihiro Kawaoka, University of Wisconsin, Madison, 1:1000 dilution), Rab11 (BD Biosciences, Franklin Lakes, NJ, USA; 1:1000 dilution), GFP (cl. GF28R; Thermo Fisher Scientific; 1:1000 dilution), and rabbit polyclonal antibody against α-tubulin (Medical & Biological Laboratories, Nagoya, Japan; 1:1000 dilution). After incubation with horseradish-peroxidase-labeled secondary antibody staining using anti-mouse IgG (Cell Signaling Technology, Danvers, MA, USA; 1:10,000 dilution) or anti-rabbit IgG (Cell Signaling Technology; 1:5000 dilution) for 1 h at room temperature, the bound antibodies were visualized using Clarity Wester ECL Substrate (Bio-Rad Laboratories, Hercules, CA, USA) in an ATTO LuminoGraph II (ATTO, Tokyo, Japan). Western blot band intensity was quantified using the CSAnalyzer4 software (ver. 2.4.3.ATTO).

### 2.4. Immunofluorescence Staining

HEK293 cells grown in an 8-well chamber (Ibidi, Fitchburg, WI, USA) were transfected with the expression plasmids for wtVP40, HiBiT-VP40 (N), HiBiT-VP40 (C), or HiBiT-VP40 L117R using TransIT-X2. At 24 h.p.t., the cells were fixed with 4% paraformaldehyde (Electron Microscopy Sciences, Hatfield, PA, USA) in phosphate-buffered saline (PBS) for 10 min at room temperature. Following fixation, cells were permeabilized with PBS containing 0.05% Triton X–100 (Nacalai Tesque Inc., Kyoto, Japan) for 10 min and then blocked in PBS containing 2% bovine serum albumin (Nacalai Tesque Inc.) for 20 min at room temperature. The cells were incubated with mouse monoclonal antibody against EBOV VP40 (cl.3G5) for 1 h at room temperature, washed with PBS, and then incubated with Alexa Fluor 488-labeled secondary antibodies (Thermo Fisher Scientific, 1:2000 dilution) for 1 h at room temperature. Nuclei were counterstained with Hoechst 33,342 (Cell Signaling Technology). Images were acquired using a confocal laser scanning microscope with an oil immersion objective lens at a magnification of 60× (Fluoview FV3000, Evident Scientific, Tokyo, Japan) and analyzed using the FV31S-SW software (ver. 2.4.1, Evident Scientific). Total fluorescence intensity of VP40 derivatives in each cell was quantified using the ImarisCell module (IMARIS; OXFORD Instruments, Abingdon, Oxfordshire, UK).

### 2.5. Negative Stain Electron Microscopy

For preparation of Ebola VLPs, Expi293F cells were co-transfected with expression plasmids for GP, NP, and wtVP40, HiBiT-fused VP40 [either HiBiT-VP40 (N) or HiBiT-VP40 (C)], or HiBiT-VP40 L117R using TransIT-X2. At 72 h.p.t., the culture supernatant was harvested and centrifuged at 440× *g* for 5 min and then at 2380× *g* for 15 min. VLPs were precipitated through a 20% sucrose cushion via ultracentrifugation at 14,860× *g* for 1 h at 4 °C. The precipitated VLPs were suspended and fixed overnight at 4 °C in 2.5% glutaraldehyde (Electron Microscopy Sciences) prepared in 0.1 M cacodylate buffer (pH 7.4; Nacalai Tesque Inc., Kyoto, Japan). Each sample was then loaded onto a 200-mesh copper grid with a carbon-coated plastic film (Nisshin EM, Tokyo, Japan) immediately following glow discharge and negatively stained with uranyl acetate solution (1%, *w*/*v*) for 15 s. The morphology of each sample was examined using a JEM-1400Flash transmission electron microscope (JEOL, Tokyo, Japan) at an acceleration voltage of 80 kV.

### 2.6. HiBiT Assay

HEK293 cells were transfected with the expression plasmids for wtVP40, HiBiT-VP40 (N), HiBiT-VP40 (C), or HiBiT-VP40 L117R using TransIT-X2. At 48 h.p.t., the supernatants were loaded to a 96-well black plate, and nano-luciferase activity was examined using a Nano-Glo^®^ HiBiT Extracellular Detection System or Nano-Glo^®^ HiBiT Lytic Detection System (Promega, Madison, WI, USA) according to the manufacturer’s instructions. To determine the cellular expression of HiBiT-fused VP40, the cells were washed and resuspended in PBS, and an equivalent volume of Nano-Glo HiBiT Lytic Reagent was added. The mixtures were loaded into a 96-well black plate, and luciferase activity was measured using the Nano-Glo^®^ HiBiT Lytic Detection System according to the manufacturer’s instructions.

### 2.7. Effect of Rab11 Downregulation and Dominant-Negative Rab11 on HiBiT-Fused VP40 Production

HEK293 cells were transfected with siRNAs targeting human Rab11a and Rab11b using TransIT-X2. As a control, cells were transfected with a non-targeting siRNA (Thermo Fisher Scientific). To determine the effect of dominant-negative Rab11 expression (dnRab11) on HiBiT-VP40 production, HEK293 cells were transfected with expression plasmids encoding GFP-wtRab11 or -dnRab11 using TransIT-X2. pCAGGS-transfected cells served as an additional control. At 24 h.p.t, cells were transfected with HiBiT-VP40 (N). At 48 h.p.t, expression of GFP-Rab11 was analyzed using Western blotting. HiBiT-VP40 (N) expression in the supernatant and cell lysate was analyzed using Western blotting and HiBiT assays, as described above. The effect of GFP-Rab11 on the distribution of VP40 derivatives was determined using immunofluorescence staining, as described above. Total fluorescence intensity of VP40 derivatives in each cell was quantified, as described above.

### 2.8. Inhibitor Treatment

HEK293 cells were transfected with an expression plasmid for HiBiT-VP40 (N). At 4 h.p.t, the cells were treated with or without serial two-fold dilutions of nocodazole (Sigma-Aldrich). Dimethyl sulfoxide (DMSO; Sigma-Aldrich) was used as a solvent control. HiBiT-VP40 (N) expression in the supernatant and VLP-producing cells was analyzed using Western blotting or HiBiT assays, as described above. Cell viability was assessed using a Cell Counting Kit–8 (Dojindo, Kumamoto, Japan). The relative luciferase activity and cell number were calculated by setting the value of untreated cells to 100%. The relative luminescence of the total cell lysate (TCL) or supernatant (Sup) was normalized to the relative cell number. The effect of nocodazole on the distribution of VP40 derivatives was determined using immunofluorescence staining as described above. Total fluorescence intensity of VP40 derivatives in each cell was quantified as described above.

### 2.9. Statistical Analysis

Western blot analysis and luciferase assays were performed in three independent experiments. Statistical analysis was performed using one-way analysis of variance (ANOVA) or Student’s *t*-test with the GraphPad Prism 9.5.1 software.

## 3. Results

### 3.1. Establishment and Characterization of HiBiT-VP40

**Figure 1:**
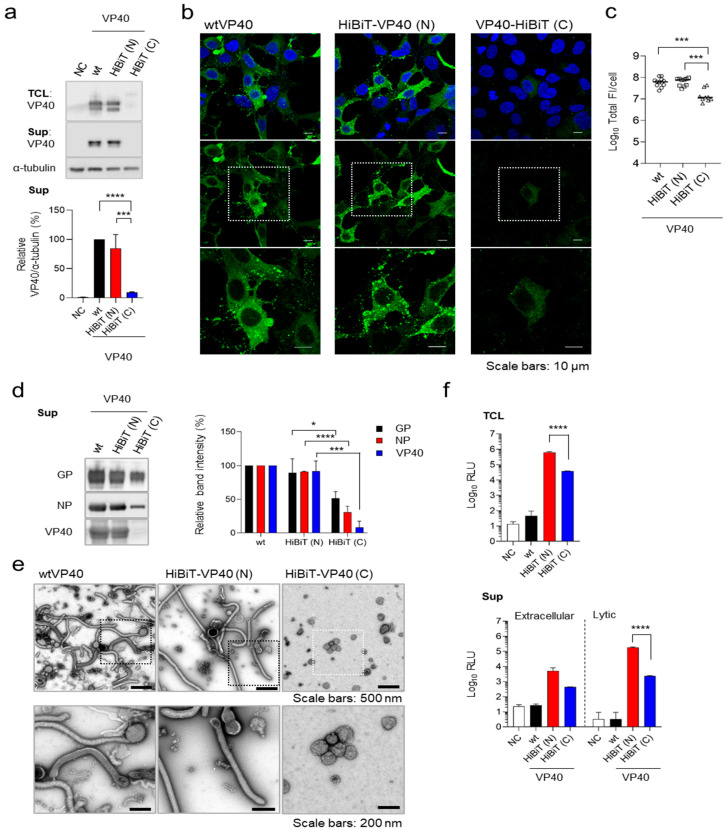
Establishment of HiBiT-fused VP40. (**a**) Western blot analysis of HiBiT-VP40. HEK293 cells (2.5 × 10^5^ cells) were transfected with expression plasmids for wtVP40, HiBiT-fused at the N- [HiBiT-VP40 (N)] or C-terminus of VP40 [HiBiT-VP40 (C)]. VP40 expression in total cell lysate (TCL; top) and cell culture supernatant (sup; middle) was analyzed using Western blotting. The intensities of the bands corresponding to VP40 were quantified. VP40 expression in TCL derivatives was normalized to that of α-tubulin. (**b**,**c**) Characterization of the intracellular distribution of HiBiT-VP40. HEK293 cells (5 × 10^4^ cells) were transfected with each expression plasmid. At 24 h post-transfection, the cells were harvested, and the distribution of wtVP40, HiBiT-VP40 (N), or HiBiT-VP40 (C) was analyzed using immunofluorescence staining. Nuclei were counterstained with DAPI. High-magnification images are shown at the bottom (insets); scale bars: 10 µm (**b**). Quantification of fluorescence intensities of expressed proteins, where the total fluorescence intensities (Total FI) of individual VP40 derivative-positive cells were quantified (**c**). (**d,e**) Morphological characterization of VLPs. Expi293F cells (7.5 × 10^7^ cells) were co-transfected with expression plasmids for GP, NP, and individual VP40 derivatives. At 72 h post-transfection, the supernatants were harvested, and VLPs were purified using ultracentrifugation. Purified VLPs derived from the cells expressing wtVP40, HiBiT-VP40 (N), or HiBiT-VP40 (C) were subjected to Western blotting (**d**, left) and negative-staining electron microscopy (**e**). The intensities of the bands corresponding to GP, NP, and VP40 were quantified (**d**, right). High-magnification images are shown at the bottom (insets) (**e**). Scale bars: 200 or 500 nm. (**f**) Luciferase activity of HiBiT-fused VP40 derivatives expressing cells and supernatants. HEK293 cells (2.5 × 10^5^ cells) were transfected with each plasmid. At 48 h post-transfection, the cells and supernatant were harvested. Luminescence activity (Luminescence; RLU) of the cell lysates was measured using the Nano-Glo HiBiT lytic detection system (top). Supernatant RLU was measured using either the Nano-Glo HiBiT extracellular detection system (bottom, left) or Nano-Glo HiBiT lytic detection system (bottom, right). The relative band intensities (**a**,**d**) were calculated by setting the value of wtVP40-expressing cells to 100%. The experiment was performed three times independently. Representative blots (**a**,**d**) and images (**b**,**e**) are shown. Data (**a**,**c**,**f**) are presented as the mean ± SD. *, *p* < 0.05; ***, *p* < 0.001; ****, *p* < 0.0001 vs. respective control (one-way ANOVA).

To enable the detection of EBOV VLP formation, we developed a HiBiT-based detection system. To establish a detection system for the released VLPs, we constructed expression plasmids for EBOV VP40 fused with HiBiT at its N- or C-terminus. The effect of fusion of HiBiT on VP40 expression and function was evaluated by transiently expressing these constructs in HEK293 cells. Both wild-type (wt)VP40 and HiBiT-VP40 (N) were detected as a doublet. Based on previous studies [16,31], we speculated that the doublet band observed likely reflects the phosphorylation status. The upper-to-lower band intensity ratio was consistently similar between wtVP40 and HiBiT-VP40 (N), indicating that the phosphorylation status of VP40 was largely unaffected by fusion of HiBit-tag. HiBiT-VP40 (N) was expressed at levels comparable to those of wild-type VP40 (wtVP40) in cells and efficiently produced VLPs in the supernatant (Figure 1a). In contrast, HiBiT-VP40 (C) exhibited abnormal molecular sizes in cell lysates, and the expression level of HiBiT-VP40 (C) was nearly undetectable in the supernatant (Figure 1a).

Next, we examined the subcellular localization of HiBiT-VP40. wtVP40 was diffusely localized in the cytoplasm and nucleus while also forming distinct clusters in the plasma membrane (PM) (Figure 1b, left). HiBiT-VP40 (N) exhibited a similar distribution pattern to that of wtVP40 (Figure 1b, middle). In contrast, transiently expressed HiBiT-VP40 (C) was diffusely distributed in the cytoplasm and nucleus but did not form clusters in the PM (Figure 1b, right). Consistent with the results of Western blotting (Figure 1a), the total fluorescence intensities of HiBiT-VP40 (C)-positive cells were significantly lower than those of wtVP40 and HiBiT-VP40 (N) (Figure 1c).

To confirm that fusion of HiBiT to VP40 does not affect EBOV particle formation or its incorporation into EBOV VLPs, we generated VLPs by expressing the major EBOV structural proteins GP, NP, and either wild-type or HiBiT-fused VP40 in Expi293F cells. Western blotting analysis confirmed that HiBiT-VP40 (N) was incorporated into VLPs at levels comparable to those of wtVP40 (Figure 1d). In contrast, HiBiT-VP40 (C) showed substantially lower incorporation into VLPs, with a band intensity barely detectable compared with that of wtVP40 (Figure 1d). We then analyzed the morphological properties of the EBOV VLPs using transmission electron microscopy. VLPs released from HiBiT-VP40 (N)-expressing cells displayed filamentous morphologies, resembling those released by cells co-expressing wtVP40 (Figure 1e left and middle). In contrast, the number of VLPs released from HiBiT-VP40 (C)-expressing cells was reduced, and the VLPs exhibited abnormal structural features (Figure 1e, right).

We then assessed HiBiT expression and VLP release by measuring luminescence activity in HiBiT-VP40-expressing cells or their supernatants. Cells and their supernatants were collected separately from individual HiBiT-VP40-expressing cells and treated with two independent detection reagents. The extracellular system is designed to detect HiBiT-tagged proteins on the cell surface or secreted into the extracellular space using a non-lytic reagent. Conversely, the lytic system is used to detect HiBiT-tagged proteins within cells or membranous particles by disrupting the lipid bilayers to release the HiBiT tag and allow it to interact with the complementary LgBiT subunit. High luciferase activity was detected in the lysates of HiBiT-VP40 (N)-expressing cells (Figure 1f, top). The HiBiT activity in the lysate of HiBiT-VP40 (C)-expressing cells was approximately 12-fold lower compared with that in HiBiT-VP40 (N), suggesting that fusion of the HiBiT tag to the C-terminus might negatively impact the expression level of VP40. As VP40 is incorporated in the viral particles, the HiBiT fused to VP40 in the supernatants is not accessible to LgBiT using an extracellular detection reagent, leading to the low level of luciferase activity (Figure 1f, bottom, left). In contrast, the luciferase activity increased when the supernatants from HiBiT-VP40 (N)-expressing cells were lysed, suggesting that HiBiT-VP40 (N) was incorporated into VLPs (Figure 1f, right). The luciferase activity in the supernatant from HiBiT-VP40 (C)-expressing cells was approximately 77-fold lower than that from HiBiT-VP40 (N) (Figure 1f, bottom, right), which is consistent with the reduction in VLP production determined by Western blotting (Figure 1d). These findings indicate that HiBiT fusion at the N-terminus retained VP40′s function. Therefore, HiBiT-VP40 (N) was selected for further characterization.

**Figure 2:**
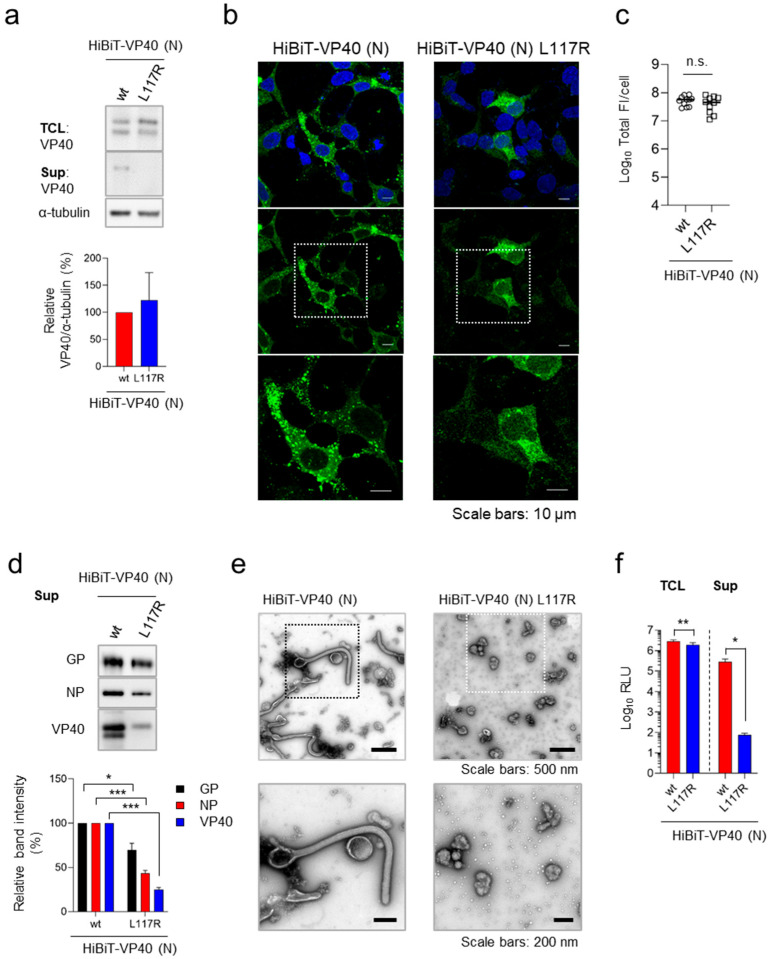
Validation of HiBiT assay using budding defective VP40 mutant. (**a**) Western blot analysis of HiBiT-fused VP40 derivatives. HEK293 cells (2.5 × 10^5^ cells) were transfected with expression plasmids for HiBiT-VP40 (N) or -VP40 L117R. VP40 expression in total cell lysate (TCL; top) and cell culture supernatant (Sup; middle) was analyzed. The intensities of the bands corresponding to VP40 were quantified. VP40 expression in TCL derivatives was normalized to that of α-tubulin. (**b**,**c**) Characterization of the intracellular distribution of HiBiT-VP40 derivatives. HEK293 cells (5 × 10^4^ cells) were transfected with expression plasmids for HiBiT-VP40 (N) or -VP40 L117R. At 24 h post-transfection, the cells were harvested, and the distribution of HiBiT-VP40 derivatives was analyzed using immunofluorescence staining. Nuclei were counterstained with DAPI. High-magnification images are shown at the bottom (insets); scale bars: 10 µm (**b**). Quantification of fluorescence intensities of expressed proteins, where total fluorescence intensities (Total FI) of individual VP40 derivative-positive cells were quantified (**c**). (**d**,**e**) Morphological characterization of VLPs. Expi293F cells (7.5 × 10^7^ cells) were co-transfected with expression plasmids for GP, NP, and HiBiT-VP40 (N) or -VP40 L117R. After 72 h post-transfection, the supernatants were harvested and purified as described elsewhere. The VLPs were analyzed by Western blotting (**d**, top) and subjected to negative-staining electron microscopy (**e**). The intensities of the bands corresponding to GP, NP, and VP40 were quantified (**d**, bottom). High-magnification images are shown at the bottom (insets). Scale bars: 200 or 500 nm. (**f**) Luciferase activity of HiBiT-fused VP40 derivative-expressing cells and supernatants. HEK293 cells (2.5 × 10^5^ cells) were transfected with each expression plasmid. At 48 h post-transfection, the cells and supernatant were harvested, and luminescence activity was measured as described elsewhere. The relative band intensities (**a**,**d**) were calculated by setting the value of HiBiT-VP40-expressing cells to 100%. The experiment was performed three times independently, and the representative blot (**a**,**d**), images (**b**,**e**), and data (**c**,**f**) are shown. Data are presented as the mean ± SD. *, *p* < 0.05; **, *p* < 0.01; ***, *p* < 0.001 vs. respective control (one-way ANOVA). n.s., not significant vs. respective control (Student’s *t*-test).

To further validate the specificity of HiBiT-VP40 (N) including VLP, we analyzed a VP40 mutant, VP40 L117R, which lacks the ability to traffic to the plasma membrane and subsequently form VLPs [32]. Western blotting analysis confirmed that HiBiT-VP40 L117R was expressed at levels comparable to those of HiBiT-VP40 (N) in transfected cells (Figure 2a, top and quantitative data). In contrast, HiBiT-VP40 L117R was not detected in the supernatant (Figure 2a, middle). Immunofluorescence staining analysis revealed a lack of formation of a filamentous morphology from the surface of cells expressing HiBiT-VP40 L117R (Figure 2b). The total fluorescence intensities of HiBiT-VP40 L117R-positive cells were similar to those of wtVP40 (Figure 2c). Western blotting analysis revealed that there was a lower incorporation of HiBiT-VP40 L117R in the VLPs (Figure 2d), which was consistent with the results shown in Figure 2a. Transmission electron microscopy revealed that few particles with non-filamentous morphologies were released from the cells under the expression of VP40 L117R (Figure 2e). Consistent with the data in Figure 2a–d, the supernatant of the cells expressing HiBiT-VP40 L117R exhibited significantly reduced HiBiT activity, while the intracellular expression of HiBiT-VP40 L117R was at almost the same level as that of HiBiT-VP40 (N) (Figure 2a,c,f). These results confirm that our detection system specifically reflects VLP formation and release, which are mediated by functionally competent VP40.

### 3.2. Validation of the HiBiT-VP40 System

**Figure 3:**
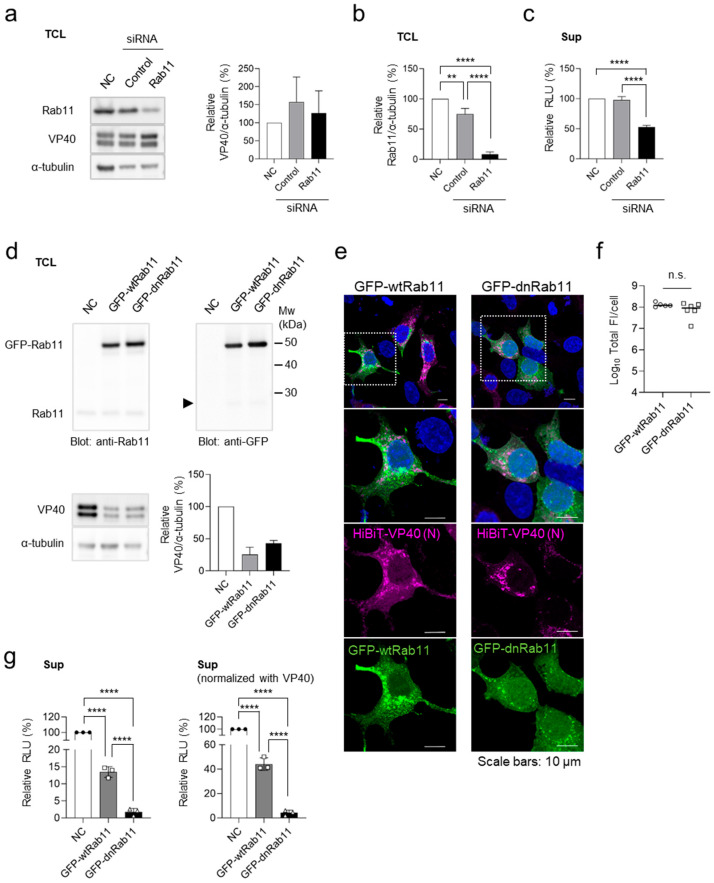
Validation of the HiBiT-VP40 detection system. (**a**–**c**) Effect of *Rab11* downregulation on VLP formation. HEK293 cells (2.5 × 10^5^ cells) were treated with control siRNA or siRNAs against Rab11a and Rab11b. Untreated cells served as the negative control (NC). At 24 h.p.t., cells were transfected with an expression plasmid for HiBiT-VP40 (N). At 48 h.p.t., expression of Rab11 or HiBiT-VP40 (N) in each cell lysate were determined using Western blot analysis (**a**). The intensities of the bands corresponding to VP40 (**a**, right) and Rab11 (**b**) were normalized to that of α-tubulin. The luciferase activity of each cell supernatant was measured using a Nano-Glo HiBiT lytic detection system (**c**). The relative band intensities (**a,b**) or luminescence (RLU; **c**) were calculated by setting the value of siRNA-untreated HiBiT-VP40-expressing cells to 100%. (**d**–**g**) Effect of the dominant-negative form of Rab11 on VLP formation. HEK293 cells (2.5 × 10^5^ cells) were transfected with expression plasmids for GFP-wtRab11 or GFP-dnRab11. As a control, backbone plasmid-transfected cells were used (NC). At 4 h.p.t., cells were transfected with expression plasmid for HiBiT-VP40 (N). At 48 h.p.t., the expression levels of Rab11, GFP, or HiBiT-VP40 (N) in each cell lysate were determined using Western blot analysis (**d**). Arrows: breakdown products of GFP (27 kDa). The intensities of the bands corresponding to VP40 were normalized to that of α-tubulin. The relative band intensities were calculated by setting the value of backbone plasmid-transfected cells to 100% (**d**, bottom, right). HEK293 cells (5 × 10^4^ cells) were transfected with expression plasmids for GFP-wtRab11, or GFP-dnRab11. At 4 h.p.t., cells were transfected with expression plasmid for HiBiT-VP40 (N). At 24 h post-transfection, the cells were harvested, and the distribution of HiBiT-VP40 was analyzed using immunofluorescence staining. Nuclei were counterstained with DAPI. High-magnification images are shown at the bottom (insets); scale bars: 10 µm (**e**). Quantification of fluorescence intensities of expressed proteins, where the total fluorescence intensity (Total FI) of HiBiT-VP40 (N)- and GFP-Rab11 derivative-positive cells was quantified (**f**). The luciferase activity of each cell supernatant was measured using a Nano-Glo HiBiT lytic detection system. Relative RLU was calculated by setting the value of the control cells (**g**, left) and further normalized with VP40 (**g**, right) to 100%. The experiment was performed three times independently, and the representative blot (**a**,**d**), images (**e**), and data (**b**–**g**) are shown. Data are presented as the mean ± SD. **, *p* < 0.01, ****, *p* < 0.0001 vs. respective control (one-way ANOVA). n.s., not significant vs. respective control (Student’s *t*-test).

We previously demonstrated that the small GTPase Rab11 plays a role in VP40-mediated VLP release by regulating recycling endosome trafficking [32]. To validate the HiBiT-VP40 system, we investigated the effect of Rab11 downregulation on HiBiT activity in VLP-producing cells. Western blot analysis confirmed the effective downregulation of Rab11 in HEK293 cells treated with siRNAs targeting two Rab11 isoforms: Rab11a [33] and Rab11b [34] (Figure 3a,b). The cells were transfected with the expression plasmid for HiBiT-VP40 (N). Although Rab11 knockdown had a minimal effect on the expression of HiBiT-VP40 (N) (Figure 3a), it significantly reduced the luciferase activity in the cell supernatants (Figure 3c), indicating impaired VLP release.

We next examined the effect of the dominant-negative form of Rab11 (dnRab11), which lacks GTPase activity. Cells were transfected with expression plasmids of the GFP-fused wild-type Rab11 (GFP-wtRab11) or dnRab11 (GFP-dnRab11), followed by transfection with the HiBiT-VP40 (N) expression plasmid. Co-expression of GFP-fused Rab11 derivatives partially reduced HiBiT-VP40 (N) expression compared with that in the control (Figure 3d). The effect of the Rab11 derivatives on the distribution of VP40 was also examined using immunofluorescence staining. Upon GFP-dnRab11 expression, VP40 formed aggregates in the cytoplasm without an intense distribution in the PM (Figure 3e). The total fluorescence intensities of HiBiT-VP40-positive cells were comparable between GFP-wt-Rab11 and GFP-dnRab11 (Figure 3f). Despite the reduction in HiBiT-VP40 (N) expression, both the actual (Figure 3g left) and normalized (Figure 3g right) HiBiT activity in the supernatants of the cells expressing dnRab11 significantly reduced compared with that of wtRab11-transfected cells. These results further validate the evaluation system for VLP formation.

### 3.3. Application of HiBiT-VP40 (N) for Evaluation for the Effect of Inhibitors of VLP Formation

Previous reports have indicated that VP40-mediated VLP formation depends on the cytoskeleton network [35,36]. To assess the utility of the HiBiT system for screening inhibitors targeting VP40-mediated viral particle formation, we evaluated the effect of nocodazole, a microtubule depolymerizing agent known to inhibit VLP release [32,35,37].

**Figure 4:**
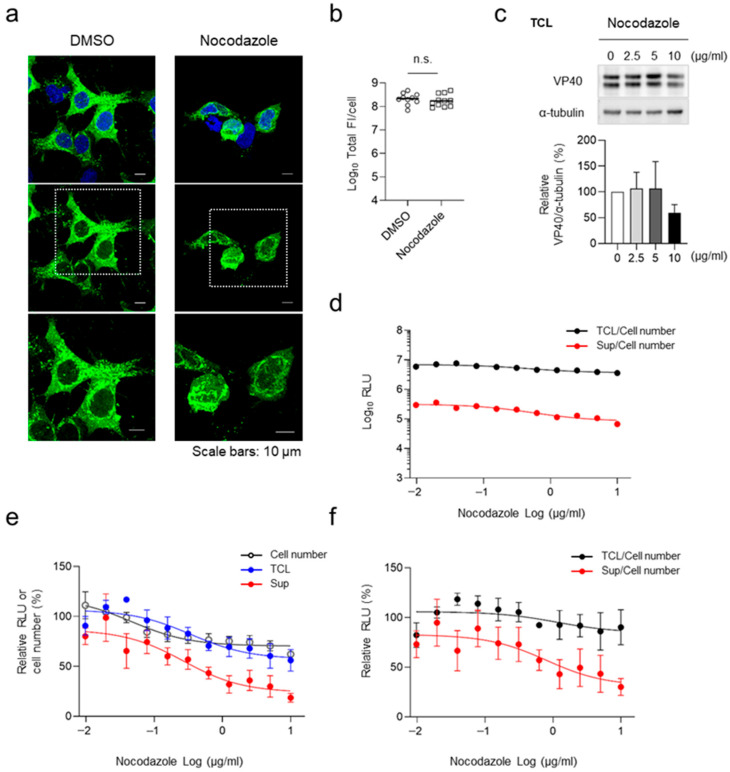
Effect of nocodazole treatment on VLP formation. (**a**) Characterization of the intracellular distribution of HiBiT-VP40 in nocodazole-treated cells. HEK293 cells (5 × 10^4^ cells) were transfected with expression plasmids for HiBiT-VP40 (N). At 4 h.p.t, the cells were treated with 2.5 µg/mL nocodazole. At 20 h post-transfection, the cells were harvested, and the distribution of HiBiT-VP40 derivatives was analyzed using immunofluorescence staining. Nuclei were counter-stained with DAPI. High-magnification images are shown at the bottom (insets). Scale bars: 10 µm. Quantification of fluorescence intensities of expressed proteins, where the total fluorescence intensity (Total FI) of HiBiT-VP40 (N)-positive cells was quantified (**b**). (**c**) Western blot analysis of HiBiT-fused VP40. HEK293 cells (2.5 × 10^5^ cells) were transfected with expression plasmids for HiBiT-VP40 (N). At 4 h post-transfection, the cells were treated with nocodazole at the indicated concentrations. VP40 expression in total cell lysate (TCL; top) was analyzed. The intensities of the bands corresponding to VP40 was normalized to that of α-tubulin (bottom). (**d–f**) Effect of nocodazole on VLP formation. HEK293 cells (2.5 × 10^5^ cells) were transfected with an expression plasmid for HiBiT-VP40 (N). At 4 h.p.t., cells were treated with nocodazole at the indicated concentrations. At 48 h.p.t., the luciferase activity of each cell supernatant was measured. Cell number was analyzed using a Cell Counting Kit–8. The absolute luminescence intensities are shown (**d**). The relative band intensities (**c**), RLU, or cell number were calculated by setting the value of untreated cells to 100% (**e**). The relative RLU value of TCL or Sup was normalized with the relative cell number value (**f**). The experiment was performed three times independently, and the representative images (**a**) and blot (**c**) are shown. Data are presented as the mean ± SD. n.s. not significant vs. respective control (Student’s *t*-test).

HEK293 cells were transfected with the expression plasmid for HiBiT-VP40 (N). At 4 h.p.t., cells were treated with nocodazole at various concentrations. Immunofluorescence staining analysis revealed that cluster formation of HiBiT-VP40 (N) in the cell periphery was suppressed under nocodazole treatment (Figure 4a). The release of HiBiT-VP40 (N) was suppressed in the supernatant in a dose-dependent manner (Figure 4d–f), with minimal effects on cell number and cellular expression of HiBiT-VP40 (N) (Figure 4b,c,e). Based on the results of absolute luminescence intensities, approximately 5% of the cellular HiBiT VP40 was released into the supernatant in the VLPs (Figure 4d), indicating that suppression of the release of VLPs may not impact the amount of cellular HiBiT-VP40 because the cells retained abundant intracellular VP40. These results suggest that the HiBiT-based assay system developed in the present study is applicable for the evaluation of inhibitors targeting EBOV particle formation.

## 4. Discussion

Handling infectious EBOV requires BSL-4 laboratory facilities, which are limited in number worldwide. Therefore, developing highly sensitive and convenient detection assays that can be performed under lower-biosafety-level conditions is crucial for antiviral compound screening against EBOV. Previous studies have used computational or luciferase-based screening methods to identify several compounds targeting VP40 [21,22,23,24,25]. A reporter-based assay has been reported to evaluate VLP formation by measuring the incorporated luciferase in the viral particles [24,25], although this method may not directly reflect VP40-mediated budding activity. Furthermore, a previous screening study utilized fluorescent protein (27 kDa)-fused VP40 to identify inhibitors associated with viral particle assembly sites within the host PM [26]. In addition, a cell-based assay to quantify VP40-mediated VLP release by directly fusing the relatively large NanoLuc protein (~19 kDa) to VP40 was reported [27]. However, fusion of these large proteins may interfere with intramolecular interactions of VP40.

Nonetheless, for the development of novel therapeutics, a highly sensitive screening approach for direct evaluation of efficacy of the compounds on the VP40-mediated budding process is also essential. In this study, we developed a novel detection system for EBOV VP40 that operates in a BSL-2 environment, enabling its application in compound screening. In comparison with existing evaluation systems, this system provides high sensitivity and quantitative analysis by using the HiBiT tag, consisting of only 11 amino acids, allowing for the evaluation of VLP production without interfering with the self-assembly of VP40. We constructed an expression plasmid encoding VP40 fused with HiBiT at the N- or C-terminus and evaluated their expression, VLP release, and morphological properties. Our findings demonstrated that HiBiT VP40 (N) retained a similar intracellular distribution, efficiency of VLP formation, and morphology of VLPs to wtVP40 (Figure 1a–e). Furthermore, we detected high luciferase activity from VLPs using the HiBiT lytic detection system (Figure 1f). These results suggest that HiBiT fusion at the N-terminus of VP40 has minimal effect on the self-assembly process of VP40 [20]. Finally, we demonstrated that this system has potential applicability in high-throughput drug screening assays targeting the VP40-dependent viral budding process (Figure 4). In conclusion, we have established a novel evaluation system for VP40, providing a convenient platform for screening antiviral drugs that target late-stage EBOV infection, including viral particle assembly and release. Further verification of the screening results with infectious EBOV is needed. Additionally, by applying this system to VP40 derived from other pathogenic filoviruses—including other EBOV species and the Marburg virus—the system can facilitate the discovery and development of novel pan-therapeutic agents against filoviruses.

## Data Availability

The data presented in this study are available on request from the corresponding author.

## Abbreviations

The following abbreviations are used in this manuscript:

EBOV	Ebola virus
EVD	Ebola virus disease
BSL	Biosafety level
DMEM	Dulbecco’s modified Eagle’s medium
HiBiT-VP40 (N)	Fusion of HiBiT tag to EBOV VP40 at the N-terminus
HiBiT-VP40 (C)	Fusion of HiBiT tag to EBOV VP40 at the C-terminus
siRNA	Small interfering RNA
GFP-wtRab11	GFP-fused wild-type Rab11
GFP-dnRab11	GFP-fused dominant-negative form of Rab11
h.p.t.	Hours post-transfection
VLP	Virus-like particle
wtVP40	Wild-type VP40
RLU	Luminescence

## References

[1] Jacob S.T., Crozier I., Fischer W.A., Hewlett A., Kraft C.S., Vega M.A., Soka M.J., Wahl V., Griffiths A., Bollinger L. (2020). Ebola virus disease. Nat. Rev. Dis. Primers.

[2] Coltart C.E., Lindsey B., Ghinai I., Johnson A.M., Heymann D.L. (2017). The Ebola outbreak, 2013–2016: Old lessons for new epidemics. Philosophical Transactions of the Royal Society of London. Philos. Trans. R. Soc. B Biol. Sci..

[3] Marzi A., Feldmann H. (2024). Filovirus vaccines as a response paradigm for emerging infectious diseases. NPJ Vaccines.

[4] Liu C.H., Hu Y.T., Wong S.H., Lin L.T. (2022). Therapeutic Strategies against Ebola Virus Infection. Viruses.

[5] Baseler L., Chertow D.S., Johnson K.M., Feldmann H., Morens D.M. (2017). The Pathogenesis of Ebola Virus Disease. Annu. Rev. Pathol..

[6] Hoenen T., Groseth A., Feldmann H. (2019). Therapeutic strategies to target the Ebola virus life cycle. Nat. Rev. Microbiol..

[7] Martin B., Hoenen T., Canard B., Decroly E. (2016). Filovirus proteins for antiviral drug discovery: A structure/function analysis of surface glycoproteins and virus entry. Antivir. Res..

[8] Mulangu S., Dodd L.E., Davey R.T., Tshiani Mbaya O., Proschan M., Mukadi D., Lusakibanza Manzo M., Nzolo D., Tshomba Oloma A., Ibanda A. (2019). Controlled Trial of Ebola Virus Disease Therapeutics. N. Engl. J. Med..

[9] Ito H., Watanabe S., Takada A., Kawaoka Y. (2001). Ebola virus glycoprotein: Proteolytic processing, acylation, cell tropism, and detection of neutralizing antibodies. J. Virol..

[10] Furuyama W., Marzi A., Nanbo A., Haddock E., Maruyama J., Miyamoto H., Igarashi M., Yoshida R., Noyori O., Feldmann H. (2016). Discovery of an antibody for pan-ebolavirus therapy. Sci. Rep..

[11] Isono M., Furuyama W., Kuroda M., Kondoh T., Igarashi M., Kajihara M., Yoshida R., Manzoor R., Okuya K., Miyamoto H. (2020). A biaryl sulfonamide derivative as a novel inhibitor of filovirus infection. Antivir. Res..

[12] Edwards M.R., Pietzsch C., Vausselin T., Shaw M.L., Bukreyev A., Basler C.F. (2015). High-Throughput Minigenome System for Identifying Small-Molecule Inhibitors of Ebola Virus Replication. ACS Infect. Dis..

[13] Biedenkopf N., Hoenen T. (2017). Modeling the Ebolavirus Life Cycle with Transcription and Replication-Competent Viruslike Particle Assays. Methods Mol. Biol..

[14] Kolesnikova L., Bamberg S., Berghöfer B., Becker S. (2004). The matrix protein of Marburg virus is transported to the plasma membrane along cellular membranes: Exploiting the retrograde late endosomal pathway. J. Virol..

[15] Hoenen T., Biedenkopf N., Zielecki F., Jung S., Groseth A., Feldmann H., Becker S. (2010). Oligomerization of Ebola virus VP40 is essential for particle morphogenesis and regulation of viral transcription. J. Virol..

[16] Jasenosky L.D., Neumann G., Lukashevich I., Kawaoka Y. (2001). Ebola virus VP40-induced particle formation and association with the lipid bilayer. J. Virol..

[17] Noda T., Watanabe S., Sagara H., Kawaoka Y. (2007). Mapping of the VP40-binding regions of the nucleoprotein of Ebola virus. J. Virol..

[18] Timmins J., Scianimanico S., Schoehn G., Weissenhorn W. (2001). Vesicular release of ebola virus matrix protein VP40. Virology.

[19] Hartlieb B., Weissenhorn W. (2006). Filovirus assembly and budding. Virology.

[20] Bornholdt Z.A., Noda T., Abelson D.M., Halfmann P., Wood M.R., Kawaoka Y., Saphire E.O. (2013). Structural rearrangement of ebola virus VP40 begets multiple functions in the virus life cycle. Cell.

[21] Urata S., Omotuyi O.I., Izumisawa A., Ishikawa T., Mizuta S., Sakurai Y., Mizutani T., Ueda H., Tanaka Y., Yasuda J. (2022). Identification of novel chemical compounds targeting filovirus VP40-mediated particle production. Antivir. Res..

[22] Broni E., Ashley C., Adams J., Manu H., Aikins E., Okom M., Miller W.A., Wilson M.D., Kwofie S.K. (2023). Cheminformatics-Based Study Identifies Potential Ebola VP40 Inhibitors. Int. J. Mol. Sci..

[23] Khan S., Fakhar Z., Ahmad A. (2021). Targeting ebola virus VP40 protein through novel inhibitors: Exploring the structural and dynamic perspectives on molecular landscapes. J. Mol. Model..

[24] McCarthy S.E., Licata J.M., Harty R.N. (2006). A luciferase-based budding assay for Ebola virus. J. Virol. Methods.

[25] Li D., Chen T., Hu Y., Zhou Y., Liu Q., Zhou D., Jin X., Huang Z. (2016). An Ebola Virus-Like Particle-Based Reporter System Enables Evaluation of Antiviral Drugs In Vivo under Non-Biosafety Level 4 Conditions. J. Virol..

[26] Bennett R.P., Finch C.L., Postnikova E.N., Stewart R.A., Cai Y., Yu S., Liang J., Dyall J., Salter J.D., Smith H.C. (2020). A Novel Ebola Virus VP40 Matrix Protein-Based Screening for Identification of Novel Candidate Medical Countermeasures. Viruses.

[27] Lin A.E., Diehl W.E., Cai Y., Finch C.L., Akusobi C., Kirchdoerfer R.N., Bollinger L., Schaffner S.F., Brown E.A., Saphire E.O. (2020). Reporter Assays for Ebola Virus Nucleoprotein Oligomerization, Virion-Like Particle Budding, and Minigenome Activity Reveal the Importance of Nucleoprotein Amino Acid Position 111. Viruses.

[28] Dixon A.S., Schwinn M.K., Hall M.P., Zimmerman K., Otto P., Lubben T.H., Butler B.L., Binkowski B.F., Machleidt T., Kirkland T.A. (2016). NanoLuc Complementation Reporter Optimized for Accurate Measurement of Protein Interactions in Cells. ACS Chem. Biol..

[29] Chen W., Feng Y., Chen D., Wandinger-Ness A. (1998). Rab11 is required for trans-golgi network-to-plasma membrane transport and a preferential target for GDP dissociation inhibitor. Mol. Biol. Cell.

[30] Nanbo A., Kachi K., Yoshiyama H., Ohba Y. (2016). Epstein-Barr virus exploits host endocytic machinery for cell-to-cell viral transmission rather than a virological synapse. J. Gen. Virol..

[31] Harty R.N., Brown M.E., Wang G., Huibregtse J., Hayes F.P. (2000). A PPxY motif within the VP40 protein of Ebola virus interacts physically and functionally with a ubiquitin ligase: Implications for filovirus budding. Proc. Natl. Acad. Sci. USA.

[32] Nanbo A., Ohba Y. (2018). Budding of Ebola Virus Particles Requires the Rab11-Dependent Endocytic Recycling Pathway. J. Infect. Dis..

[33] Gromov P.S., Celis J.E., Hansen C., Tommerup N., Gromova I., Madsen P. (1998). Human rab11a: Transcription, chromosome mapping and effect on the expression levels of host GTP-binding proteins. FEBS Lett..

[34] Silvis M.R., Bertrand C.A., Ameen N., Golin-Bisello F., Butterworth M.B., Frizzell R.A., Bradbury N.A. (2009). Rab11b regulates the apical recycling of the cystic fibrosis transmembrane conductance regulator in polarized intestinal epithelial cells. Mol. Biol. Cell.

[35] Furuyama W., Yamada K., Sakaguchi M., Marzi A., Nanbo A. (2024). Marburg virus exploits the Rab11-mediated endocytic pathway in viral-particle production. Microbiol. Spectr..

[36] Ruthel G., Demmin G.L., Kallstrom G., Javid M.P., Badie S.S., Will A.B., Nelle T., Schokman R., Nguyen T.L., Carra J.H. (2005). Association of ebola virus matrix protein VP40 with microtubules. J. Virol..

[37] Noda T., Ebihara H., Muramoto Y., Fujii K., Takada A., Sagara H., Kim J.H., Kida H., Feldmann H., Kawaoka Y. (2006). Assembly and budding of Ebolavirus. PLoS Pathog..

